# Local treatment with the selective IκB kinase β inhibitor NEMO-binding domain peptide ameliorates synovial inflammation

**DOI:** 10.1186/ar1958

**Published:** 2006-05-09

**Authors:** Sander W Tas, Margriet J Vervoordeldonk, Najat Hajji, Michael J May, Sankar Ghosh, Paul P Tak

**Affiliations:** 1 Division of Clinical Immunology and Rheumatology F4-218, Academic Medical Center/University of Amsterdam, PO Box 22700, 1100 DE Amsterdam, The Netherlands; 2 School of Veterinary Medicine, Department of Animal Biology, University of Pennsylvania, 3800 Spruce Street, Philadelphia, PA 19104-6046, USA; 3 Immunobiology Section, Yale University Medical School, 300 Cedar Street, New Haven, CT 06519, USA

## Abstract

Nuclear factor (NF)-κB is a key regulator of synovial inflammation. We investigated the effect of local NF-κB inhibition in rat adjuvant arthritis (AA), using the specific IκB kinase (IKK)-β blocking NF-κB essential modulator-binding domain (NBD) peptide. The effects of the NBD peptide on human fibroblast-like synoviocytes (FLS) and macrophages, as well as rheumatoid arthritis (RA) whole-tissue biopsies, were also evaluated. First, we investigated the effects of the NBD peptide on RA FLS *in vitro*. Subsequently, NBD peptides were administered intra-articularly into the right ankle joint of rats at the onset of disease. The severity of arthritis was monitored over time, rats were sacrificed on day 20, and tissue specimens were collected for routine histology and x-rays of the ankle joints. Human macrophages or RA synovial tissues were cultured *ex vivo *in the presence or absence of NBD peptides, and cytokine production was measured in the supernatant by enzyme-linked immunosorbent assay. The NBD peptide blocked interleukin (IL)-1-β-induced IκBα phosphorylation and IL-6 production in RA FLS. Intra-articular injection of the NBD peptide led to significantly reduced severity of arthritis (*p *< 0.0001) and reduced radiological damage (*p *= 0.04). This was associated with decreased synovial cellularity and reduced expression of tumor necrosis factor (TNF)-α and IL-1-β in the synovium. Incubation of human macrophages with NBD peptides resulted in 50% inhibition of IL-1-β-induced TNF-α production in the supernatant (*p *< 0.01). In addition, the NBD peptide decreased TNF-α-induced IL-6 production by human RA synovial tissue biopsies by approximately 42% (*p *< 0.01). Specific NF-κB blockade using a small peptide inhibitor of IKK-β has anti-inflammatory effects in AA and human RA synovial tissue as well as in two important cell types in the pathogenesis of RA: macrophages and FLS. These results indicate that IKK-β-targeted NF-κB blockade using the NBD peptide could offer a new approach for the local treatment of arthritis.

## Introduction

Rheumatoid arthritis (RA) is a chronic inflammatory disease predominantly affecting the joints [1]. Many different cell types have been described as contributing to both the initiation phase of the disease and the chronic perpetuation of synovial inflammation. In rheumatoid synovium, the intimal lining layer shows marked hyperplasia, mainly due to expansion of intimal macrophages and fibroblast-like synoviocytes (FLS) [2]. Macrophages appear to play a pivotal role in the pathogenesis of RA because they are present in high numbers in RA synovial tissue and clearly show signs of activation, including enhanced expression of cellular surface markers like major histocompatibility complex class II molecules, pro-inflammatory cytokines such as tumor necrosis factor-α (TNF-α) [3], chemokines, and matrix metalloproteinases [4]. Furthermore, there is a highly significant positive correlation between scores for local disease activity and macrophage numbers and the expression of macrophage-derived cytokines in the synovium [5]. In addition to macrophages, other cell types, like FLS, also display altered biology. RA FLS are characterized by anchorage-independent growth and resistance to apoptosis due to constitutive activation of multiple signaling cascades (reviewed in [6,7]).

In many of the cells involved in synovial inflammation, alterations are found in intracellular signaling cascades, leading to unwanted interactions with other cells and resulting in pathology [8]. Striking abnormalities are observed in the nuclear factor (NF)-κB signal transduction pathway [9]. Phosphorylation of inhibitor of κB (IκBα) by the IκB kinase (IKK) complex is a crucial step in NF-κB/Rel activation. The IKK complex contains two catalytic subunits, named IKK-α and IKK-β, and a regulatory subunit termed NEMO (NF-κB essential modulator). NF-κB activation in response to pro-inflammatory signals is dependent mainly on IKK-β [10]. The subsequent polyubiquitination targets IκBα for degradation, releasing NF-κB dimers from the NF-κB-IκBα complex, followed by translocation to the nucleus and binding to κB enhancer elements of target genes [11].

IKK is a key convergence site of many different stimuli that induce NF-κB activation, such as pro-inflammatory cytokines and ligation of Toll-like receptors, but triggering of highly specialized antigen receptors such as the T-cell receptor is also dependent on this pathway [12]. Consequently, selective inhibition of the IKK complex has emerged as a promising strategy to block aberrant NF-κB activity in autoimmune and inflammatory diseases as well as certain cancers [13].

NF-κB is highly activated in the synovial tissue of patients with RA [14,15], with IKK-β being a key regulator of synovial inflammation [16]. Various local or systemic approaches to specifically inhibit the activation of this transcription factor by targeting the IKK complex have proven successful in the amelioration of arthritis [16-19]. Obviously, NF-κB activity is also required for normal physiology of cells or for clearing microbial pathogens, raising toxicity concerns when this pathway is blocked systemically in many different cell types at the same time. Accordingly, for development of therapies blocking NF-κB activity in RA, local intra-articular (i.a.) therapy appears more attractive.

The present study was conducted to explore the effects of specific inhibition of IKK-β-mediated NF-κB activation locally in the inflamed joint, using the well-characterized NEMO-binding domain (NBD) peptide [20]. Our data indicate that local IKK-β-targeted NF-κB blockade using a small peptide inhibitor ameliorates synovial inflammation, both in an animal model of arthritis and in human RA synovial tissue *ex vivo*, which opens up a new approach for the local treatment of RA.

## Materials and methods

### Animals

Pathogen-free male Lewis rats (150–200 g; 8–10 weeks of age at the start of the experiments) were obtained from Harlan Sprague Dawley, Inc. (Horst, The Netherlands) and were maintained in our central animal facility. The Animal Care and Use Committee of the University of Amsterdam, The Netherlands, approved all experiments.

### NBD peptides

Small-scale Fmoc (9-fluorenylmethoxycarbonyl) synthesis of the peptides was carried out on a Rainin Symphony Instrument (Rainin Instrument, LLC, Oakland, CA, USA) at the HHMI Biopolymer-Keck Foundation Biotechnology Resource Laboratory at Yale University (New Haven, CT, USA). Peptides were characterized by matrix-assisted laser desorption ionization mass spectrometry and analytical reverse-phase high-performance liquid chromatography analysis. The peptides were subsequently dissolved in dimethyl sulfoxide to a stock of 50 mM. The sequences of the wild-type and mutant (MUT) NBD peptides have been described previously [20]. The NBD peptide (3.7 kD) contains the region of IKK-β from T735 to E745 synthesized in tandem with a membrane permeabilization sequence from the drosophila antennapedia homeodomain protein. The MUT peptide (3.5 kD) is identical except that W739 and W741 are replaced by alanines to render it biologically inactive.

### Evaluation of NF-κB inhibition in FLS

Synovial biopsies were obtained by arthroscopy from different seropositive RA patients with actively inflamed joints. Human RA FLS were isolated from synovial tissue as described previously [21], grown in Dulbecco's modified Eagle's medium (DMEM)/10% fetal calf serum (FCS), and used from passages 3 to 8. For stimulation experiments, FLS were seeded onto 24-well dishes (Costar, now Corning Life Sciences, Acton, MA, USA) at 1 × 10^4 ^per well. After serum-starving for 12 hours in medium containing 0.5% FCS for synchronization, cells were pre-incubated for 2 hours with NBD or MUT peptides (50 μM) in medium containing 0.5% FCS and stimulated with IL-1-β (2.5 ng/ml). After 30 minutes of stimulation, cells were washed twice with ice-cold phosphate-buffered saline (PBS) to remove all serum proteins and then lysed in 1× SDS-PAGE sample buffer. Total intracellular protein was separated by SDS-PAGE on a 10% gel, using Rainbow-colored protein molecular weight markers (Amersham Biosciences, now GE Healthcare, Little Chalfont, Buckinghamshire, UK) as a reference, and transferred onto a polyvinylidene difluoride membrane (Bio-Rad Laboratories, Inc., Hercules, CA, USA). The membrane was blocked in Tris-buffered saline (TBS) containing 2% non-fat dry milk (Bio-Rad Laboratories, Inc.), Na_3_VO_4 _(2 mM), and 0.05% Tween 20 for 1 hour. Detection of phosphorylated (ph) and unphosphorylated proteins was performed by incubating the membranes with a primary antibody against the protein of interest overnight at 4°C. The membranes were subsequently washed and incubated with the appropriate horseradish peroxidase (HRP)-labeled secondary antibody (Bio-Rad Laboratories, Inc.) in TBS containing 2% non-fat dry milk, Na_3_VO_4 _(2 mM), and 0.05% Tween 20 for 1 hour at room temperature, and after extensive washing were assayed using the enhanced chemiluminescence detection system (GE Healthcare). Mouse monoclonal antibodies (mAbs) to ph-IκBα and total IκBα were obtained from Cell Signaling Technology, Inc. (Beverly, MA, USA). Densitometry was performed using Quantity One software (Bio-Rad Laboratories, Inc.). To study the effects of NBD on cytokine production, FLS were pre-incubated for 2 hours with NBD peptides (50 μM) and stimulated for 24 hours with recombinant human IL-1-β (2.5 ng/ml; R&D Systems, Minneapolis, MN, USA). Supernatants were harvested, and the levels of IL-6 were determined by sandwich enzyme-linked immunosorbent assay (ELISA) as described previously [22].

### AA

All rats were immunized at the base of the tail with 1 mg of Mycobacterium tuberculosis H37RA (Difco, Detroit, MI, USA) in 0.1 ml mineral oil on day 0 [16]. Clinical signs of arthritis (that is, paw swelling) were usually observed by day 10 and monitored during the course of disease by water displacement plethysmometry. Paw swelling was expressed as delta paw volume (that is, with paw volume before onset of arthritis subtracted). For i.a. treatment, the right ankle joints were injected at days 10 and 12 after immunization in animals (*n *= 6/group [dose-finding]; *n *= 15/group [clinical study]) anesthetized with isoflurane. The skin was prepared with ethanol, and NBD or MUT peptides were injected in the indicated concentrations anterolaterally into the right ankle joint in a total volume of 50 μl saline, using a 31-gauge needle on a glass syringe [23]. The course of arthritis was monitored regularly until rats were sacrificed at day 20 by CO_2 _inhalation and hind paws were collected. X-rays of the ankle joints were made, and these plain radiographs were scored for bone degradation, using a semiquantitative scoring system (demineralization [0-2+], ankle and midfoot erosions [0-2+], calcaneal erosion [0-1+], heterotopic bone formation [0-1+]; maximum possible score = 6) according to Boyle and colleagues [24].

### Immunohistochemical analysis of synovial cytokine expression

Hind paws were obtained from each rat, trimmed of skin, and fixed in 4% paraformaldehyde. After 24 hours, paraformaldehyde was replaced by 70% ethanol and x-rays of the ankle joints were made. Subsequently, the paws were decalcified for 4 weeks in decalcifying solution (15% EDTA [ethylenediaminetetraacetic acid; pH 7.5]) on a rotator at 4°C, with decalcifying solution changed twice a week. After 1 week of decalcification, the paws were longitudinally cut in half. After 4 weeks, the formalin-fixed paws were dehydrated in graded alcohol and embedded in paraffin. Paraffin-embedded paws were serially sectioned at a thickness of 4 μm. Sections were subsequently deparaffinized in xylene and rehydrated in ethanol, followed by incubation with hydrogen peroxide 30% in 0.1% Na-azide-PBS to block endogenous peroxidase activity. Antigen retrieval was obtained by boiling the sections in citrate buffer (pH 6.0) for 10 minutes.

Cytokine expression was studied by staining the sections overnight at 4°C with mAbs specific for TNF-α (10 μg/ml), IL-6 (10 μg/ml), and IL-1-β (10 μg/ml) (all from R&D Systems, Oxon, UK) in PBS/bovine serum albumin (BSA) 1%. Sections were then washed extensively and incubated with secondary HRP-conjugated swine anti-goat antibodies (Dako Denmark A/S, Glostrup, Denmark) in PBS/BSA 1% + 10% *N*-hydroxysuccinimide. Signal amplification was performed using biotinylated tyramine (PerkinElmer Life and Analytical Sciences, Boston, MA, USA) followed by streptavidine-HRP (Dako Denmark A/S) in PBS/BSA 1% as described previously [4]. Finally, peroxidase activity was detected with AEC (0.02% 3-amino-9-ethylcarbazole; Vector Laboratories, Burlingame, CA, USA) yielding red coloration. Sections were counterstained with Mayer's haemalum solution (Merck, Darmstadt, Germany) and mounted with Kaiser's glycerol gelatin (Merck) mounting medium. For quantification of cytokine expression, the sections were blinded and analyzed in a random order by computer-assisted image analysis.

### Digital image analysis

Six randomly selected fields within each section were chosen for digitizing the amount of positive signal. These images were acquired on an Olympus microscope (Olympus, Tokyo, Japan), captured using a Charged Coupled Device video camera (Sony, Tokyo, Japan), and digitized with a PV100 multimedia 16-bit color video digitizer card. In the resultant color images, the area of positive staining and the mean optical density (MOD) were measured by a macro program as described previously [25,26]. The MOD is proportional to the cellular concentration of protein. The integrated optical density is equal to the MOD multiplied by the area of positive staining.

### Culture of normal macrophages and synovial biopsies from patients with RA

For evaluating the effect of NBD on macrophage cytokine production *in vitro*, monocytes were isolated from peripheral blood of healthy controls as described previously [22] and allowed to adhere to tissue culture plastic (24-well plates; Corning Life Sciences) for 1 hour (1 × 10^6 ^cells; 1 ml). Subsequently, non-adherent cells were washed away and cells were cultured for 8–9 days to obtain macrophages, with half of the medium refreshed every 3 days [27]. Macrophages were pre-incubated for 2 hours with NBD peptides (50 μM), and cells were stimulated for 24 hours with rhIL-1-β (2.5 ng/ml; R&D Systems). Supernatants were harvested, and the levels of TNF-α were determined by sandwich ELISA as described previously [22].

To evaluate the effects of NBD on human synovial tissue, small-bore arthroscopy (2.7-mm arthroscope; Storz, Tuttlingen, Germany) was performed under local anesthesia in three patients with established, active seropositive RA [28]. The obtained biopsies (± 5 mm^3^; 3 per well; mixed locations in the joint to minimize sampling error) were cultured intact in DMEM/10% FCS in a humidified 5% CO_2 _atmosphere in the presence or absence of NBD peptides (100 μM) and after 2-hour pre-incubation stimulated with rhTNF-α (10 ng/ml; R&D Systems). After 7 days, supernatants were collected and evaluated for the presence of IL-6 by sandwich ELISA as described previously [22]. IL-6 levels were corrected for total size of the biopsies by weighing the biopsies at day 7.

### Statistical analysis

Treatment effects in the animal experiments were analyzed using repeated measures analysis of variance, with treatment and time as fixed factors and rat number as random factor. To test whether treatment-induced amelioration of arthritis in time was significant, the interaction-test treatment*time was applied (SPSS 11.5.1 Statistics, SPSS Ltd., Surrey, UK), resulting in the 'area under the curve' (AUC). Data from *in vitro *and *ex vivo *experiments were analyzed for statistical significance (GraphPad, InStat, version 2.02; GraphPad Software, Inc., San Diego, CA, USA), using the Student's *t *test or Mann-Whitney *U *test. A *p *value < 0.05 was taken as the level of significance.

## Results

### NBD peptide blocks IκBα phosphorylation and IL-6 production in RA FLS

**Figure 1:**
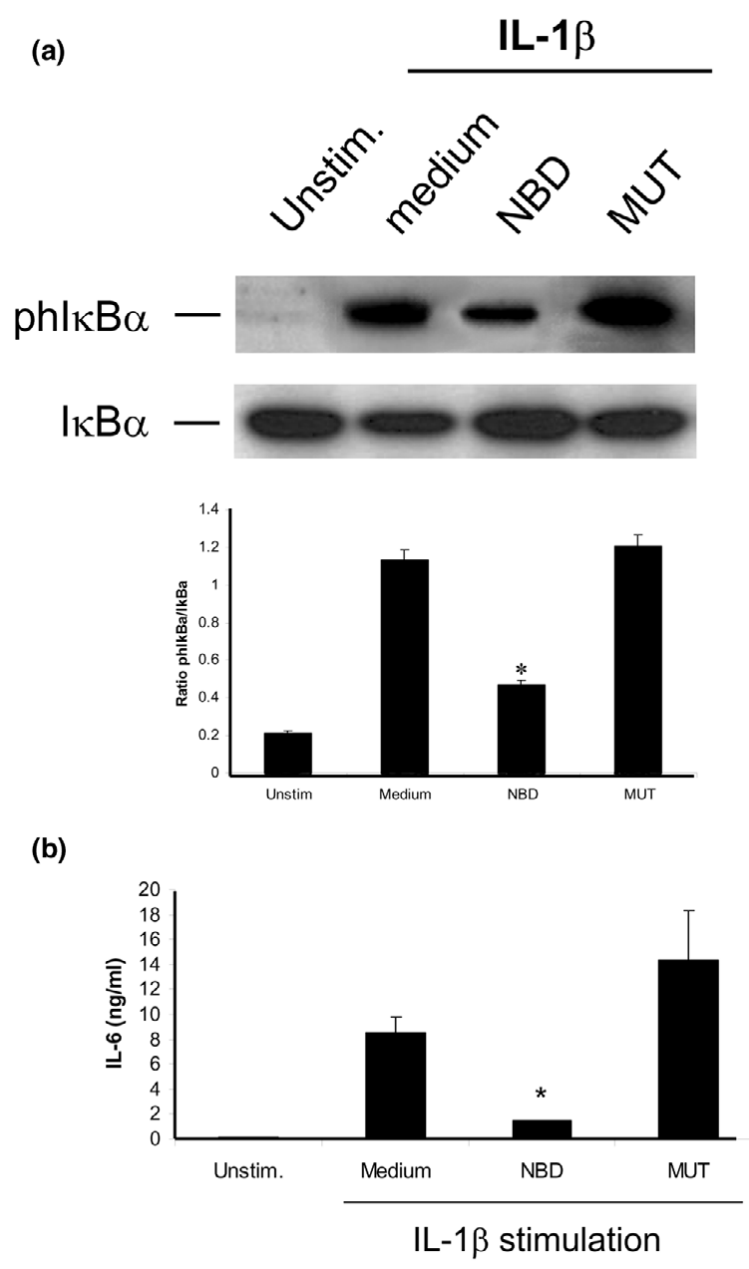
NBD peptide blocks interleukin (IL)-1-β-induced IκBα phosphorylation and IL-6 production in fibroblast-like synoviocytes (FLS). **(a)** FLS were pre-incubated with either NBD or mutant NBD (MUT) peptide at a concentration of 50 μM for 2 hours. Subsequently, cells were stimulated with IL-1-β (2.5 ng/ml) for 30 minutes, extensively washed, and lysed in sample buffer. Cell lysates were analyzed by Western blotting. One representative blot out of three is shown. Densitometry includes all three experiments, and data are expressed as mean ± standard error of the mean (*p < 0.01). **(b)** NBD peptide blocks IL-1-β-induced IL-6 production by FLS *in vitro*. FLS were pre-incubated with either NBD or MUT peptide at a concentration of 50 μM for 2 hours. Subsequently, cells were stimulated with IL-1-β (2.5 ng/ml). After 24 hours, supernatants were collected and IL-6 levels were measured by sandwich enzyme-linked immunosorbent assay. Data are representative of three independent experiments performed in triplicates (*p < 0.01).

The NF-κB blocking effect of the NBD peptide has been extensively characterized [17,18,20,29]. To evaluate the effects of IKK-β inhibition in RA FLS, we analyzed the consequences of pre-treatment with NBD on IL-1-β-induced IKK-mediated phosphorylation of IκBα as readout for NF-κB activation *in vitro*. FLS were pre-treated for 2 hours with NBD or the mutant control peptide and stimulated for 30 minutes with IL-1-β. Cells were lysed and total intracellular protein was separated using SDS-PAGE. After immunoblotting, ph- and unphosphorylated IκBα were detected using specific mAbs. NBD pre-treatment resulted in reduced IL-1-β-induced IκBα phosphorylation, whereas the mutant control peptide did not affect IκBα phosphorylation in FLS. Using densitometry, we found that the ph-IκBα/IκBα ratio was significantly reduced in NBD compared with MUT-treated FLS (*p *< 0.01). NBD treatment almost reduced the ph-IκBα/IκBα ratio to the level seen in unstimulated cells (0.47 vs. 0.21 in unstimulated cells). The mutant control peptide (MUT) did not affect IκBα phosphorylation (ph-IκBα/IκBα ratio 1.20 vs. 1.13 in stimulated control cells) (Figure 1a). The low ph-IκBα/IκBα ratio after NBD treatment of FLS was accompanied by a strong reduction in IL-1-β-induced IL-6 secretion by these cells (1.4 ± 0.1 ng/ml vs. 8.5 ± 1.3 ng/ml in stimulated cells and 14.3 ± 4 ng/ml in MUT-treated stimulated FLS, *p *< 0.01) (Figure 1b). Taken together, these data demonstrate that the NBD peptide blocks IL-1-β-induced IκBα phosphorylation in RA FLS, resulting in a less inflammatory phenotype.

### Intra-articular NBD treatment ameliorates AA in rats

**Figure 2:**
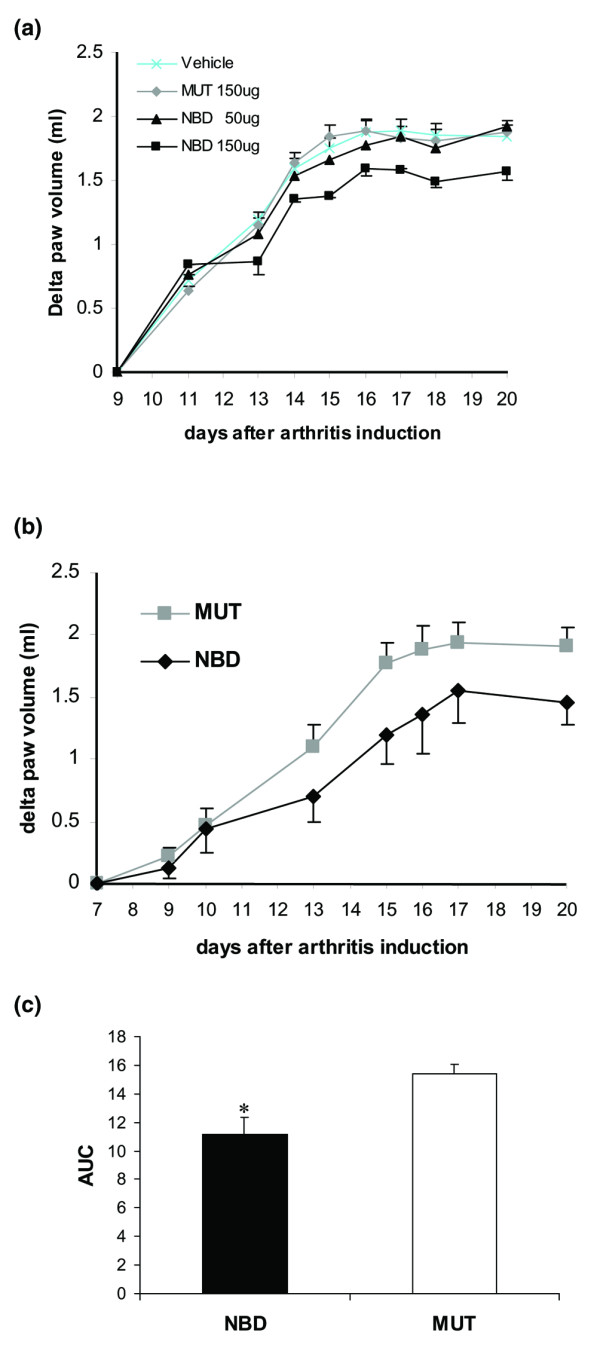
Intra-articular injection of the NBD peptide at the onset of disease ameliorates arthritis. **(a)** Dose-finding experiments. To determine the optimal dose of the NBD peptide for amelioration of arthritis, animals (*n *= 6/group) were treated intra-articularly at the onset of arthritis (day 10) and 2 days later (day 12) with either 50 or 150 μg of the peptides or vehicle. Paw swelling was measured by water displacement plethysmometry until the animals were sacrificed at day 20. Data represent mean ± standard error of the mean (SEM) paw swelling (*p *< 0.05). **(b)** To evaluate the effect of the NBD peptide on clinical arthritis compared with the mutant NBD (MUT) control peptide, animals (*n *= 15/group) were treated intra-articularly at the onset of arthritis (day 10) and 2 days later (day 12) with 150 μg of the peptides. Paw swelling was measured by water displacement plethysmometry until the animals were sacrificed at day 20. Data represent mean ± SEM paw swelling (*p *< 0.0001). (c) Results of the 'area under the curve' (AUC) calculation of the experiments displayed in (b) (*p < 0.0001).

Next, we investigated the therapeutic effects of this highly specific IKK-β inhibitor in established arthritis when administered intra-articularly. In a dose-finding study (*n *= 6/group), we found that two i.a. injections (on days 10 and 12) with a dose of 150 μg NBD peptide significantly reduced arthritis severity (*P *< 0.05), whereas a dose of 50 μg only marginally ameliorated arthritis (Figure 2a). Subsequently, we conducted a large therapeutic study in which AA was induced in rats (*n *= 15/group) on day 0. At the start of arthritis symptoms (day 10), the animals received an i.a. injection with either the NBD peptide or the MUT peptide (150 μg) into the right ankle joints. Two days later, this procedure was repeated and the course of arthritis was monitored by blinded observers until day 20 to evaluate the effects of local IKK-β inhibition on paw swelling. Intra-articular treatment with NBD resulted in significantly reduced paw swelling (AUC 11.18 ± 1.14 vs. 15.40 ± 0.70, NBD vs. MUT, respectively; *p *< 0.0001) (Figure 2b,c). Careful evaluation of the internal organs of the animals (liver, kidney, spleen, and so forth), conducted with a pathologist, did not reveal any alterations compared with the control animals. Also, no opportunistic infections occurred in the NBD-treated animals. Taken together, these findings do not suggest major systemic effects. In short, we have demonstrated in two independent AA experiments that two i.a. injections of the NBD peptide significantly ameliorated arthritis severity.

### Intra-articular NBD treatment results in reduced synovial inflammation

**Figure 3:**
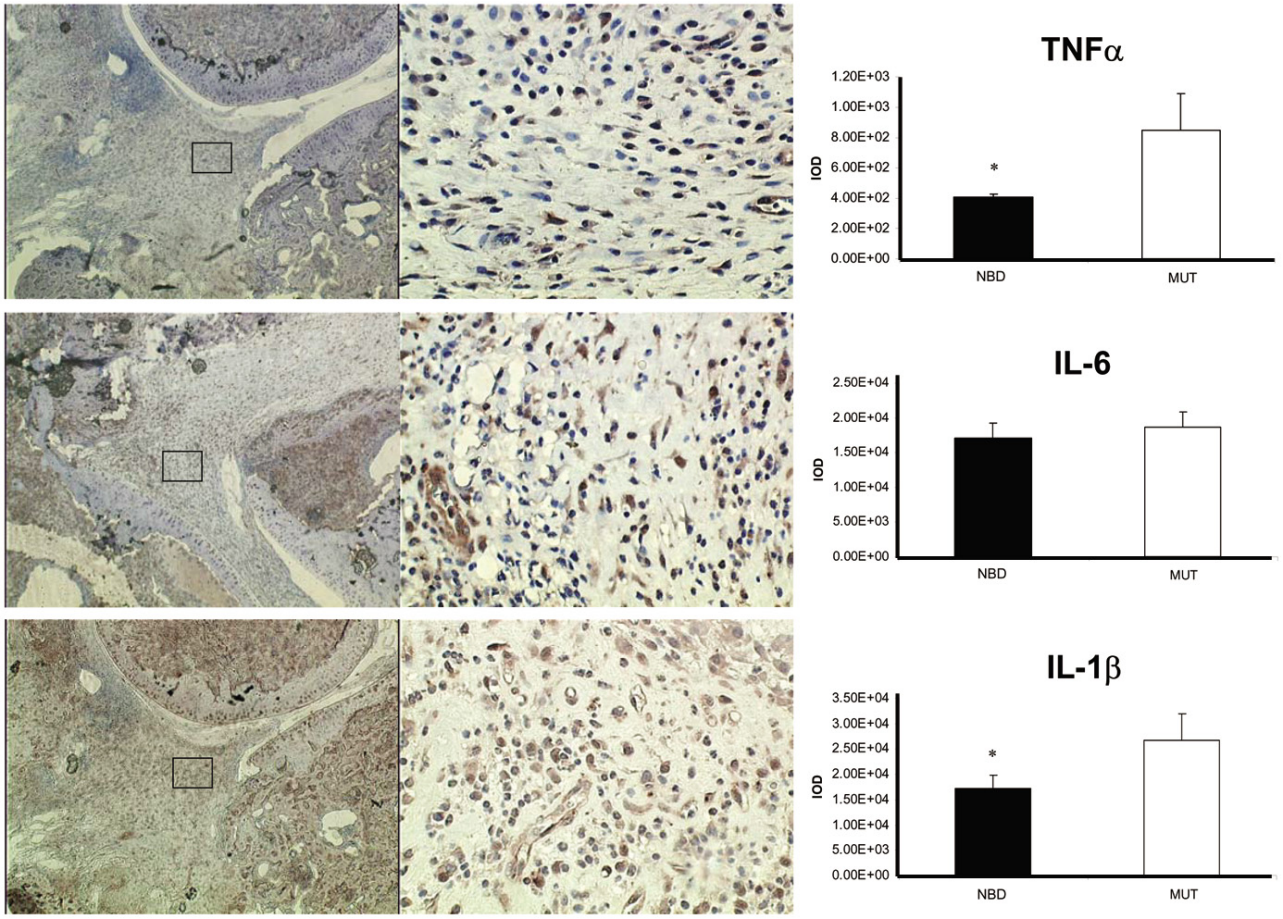
Intra-articular NBD treatment results in decreased pro-inflammatory cytokine expression. Shown are representative overview images of mutant NBD (MUT)-treated ankle joints. Expression of different cytokines was evaluated by immunohistochemical staining of paraffin-embedded ankle joints. Detailed images of synovial cytokine staining are provided (boxes in upper panels indicate synovial tissue location), followed by results from digital image analysis (*n *= 10/group). Tumor necrosis factor (TNF)-α and interleukin (IL)-1-β expression was significantly reduced in the NBD-treated group as compared with the MUT-treated group (*p = 0.05 and *p = 0.04, respectively). Data represent mean ± standard error of the mean.

Having shown the beneficial effect of the NBD peptide on the severity of arthritis, we evaluated the effects of i.a. NBD treatment on synovial cellularity *in situ*. Intra-articular injection of the NBD peptide resulted in a significant decrease of inflammatory cells in the synovial tissue compared with MUT treatment (239 ± 5 cells/mm^2 ^vs. 273 ± 7 cells/mm^2^, respectively; *p *< 0.01). Histological evaluation of NBD-treated ankle joints revealed less proliferation and invasive growth of the synovial tissue (Figure 3). Next, we evaluated the effects of the NBD peptide on synovial inflammation by performing immunohistochemical stainings on sections from paraffin-embedded rat ankle joints. Digital image analysis of comparable locations in the synovial tissue showed a clear difference between NBD- and MUT-treated animals in the expression of the pro-inflammatory cytokines TNF-α (4.06 × 10^2 ^± 2.40 × 10^1 ^versus 8.51 × 10^2 ^± 2.38 × 10^2^; *p *= 0.05) and IL-1-β (1.73 × 10^4 ^± 2.43 × 10^3 ^versus 2.66 × 10^4 ^± 5.30 × 10^3^; *p *= 0.04). IL-6 expression was not different between the two groups (1.70 × 10^4 ^± 2.17 × 10^3 ^versus 1.87 × 10^4 ^± 2.10 × 10^3^) (Figure 3).

### Intra-articular NBD treatment reduces bone destruction

**Figure 4:**
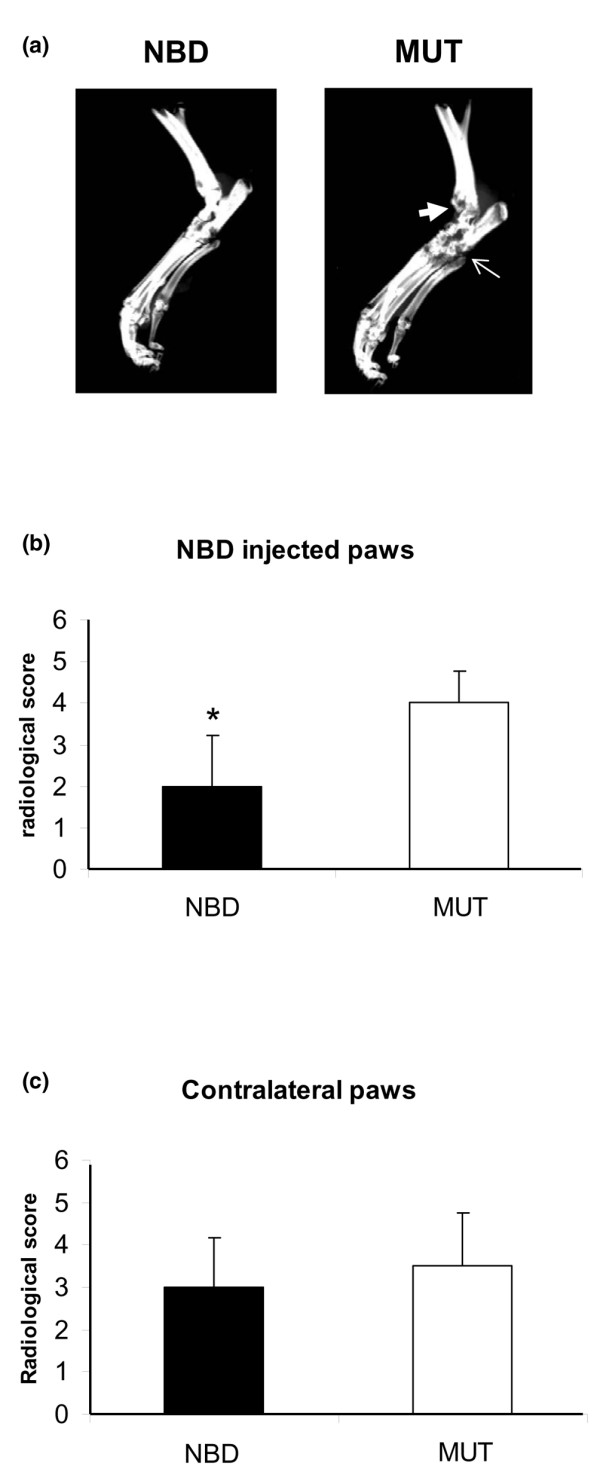
Nuclear factor-κB essential modulator binding domain (NBD) peptide treatment significantly reduces bone destruction. **(a)** X-rays of the ankle joints (*n *= 10/group) were made, and radiological damage was scored. Representative pictures for NBD- and mutant NBD (MUT)-treated ankle joints are shown. Large arrow indicates ankle demineralization. Small arrow indicates midfoot demineralization and erosions. **(b)** NBD peptide treatment significantly reduces bone destruction. Data represent mean ± standard error of the mean (SEM) radiological scores (*p < 0.04). **(c)** No significant difference in bone destruction was observed in the contralateral, non-injected paws. Data represent mean ± SEM radiological scores.

We studied the effects of NBD treatment not only on synovial inflammation, but also on bone destruction. Therefore, x-rays of the ankle joints (Figure 4a) were made, and these plain radiographs were scored for bone degradation, using a validated scoring system [24]. Intra-articular NBD treatment significantly reduced bone degradation of the injected ankle joints (*p *< 0.04) (Figure 4b) compared with MUT-treated or contralateral joints. These findings show that local IKK-β inhibition in the joint by i.a. injection of the small molecule NBD peptide not only ameliorates arthritis, but concomitantly also reduces bone destruction.

### The NBD peptide inhibits pro-inflammatory cytokine production in human macrophages and whole-tissue synovial biopsies from patients with RA

**Figure 5:**
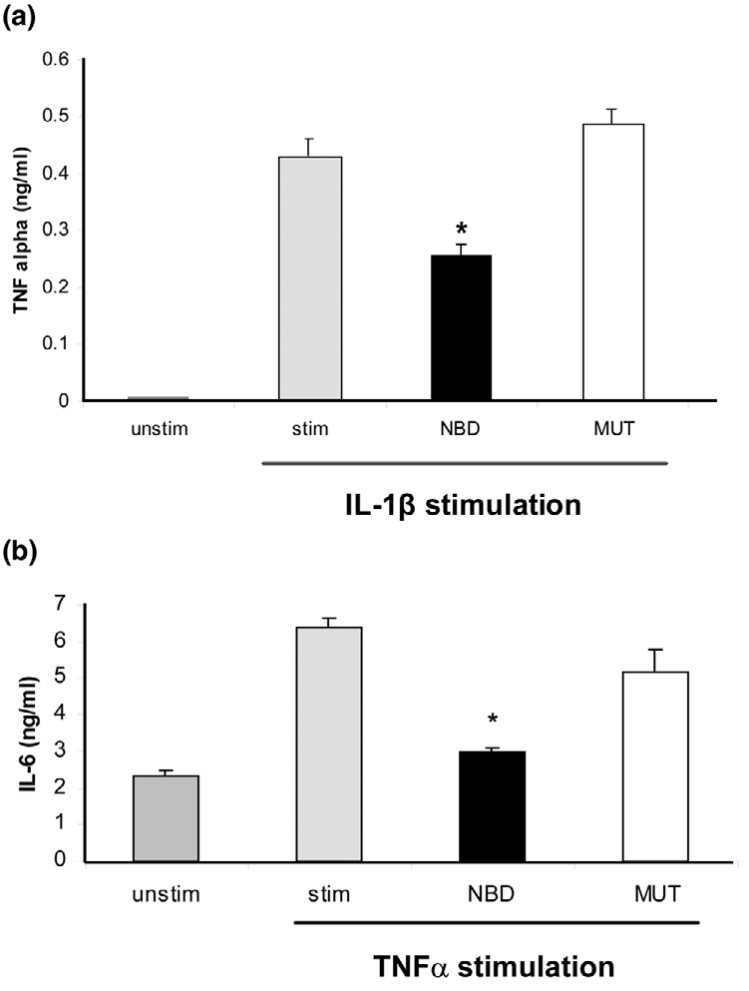
NBD peptide blocks pro-inflammatory cytokine production by human macrophages *in vitro *and rheumatoid arthritis (RA) synovial biopsies *ex vivo*. **(a)** NBD peptide blocks interleukin (IL)-1-β-induced tumor necrosis factor (TNF)-α production by human macrophages *in vitro*. Macrophages were pre-incubated with either NBD or mutant NBD (MUT) peptide at a concentration of 50 μM for 2 hours. Subsequently, cells were stimulated with IL-1-β (2.5 ng/ml). After 24 hours, supernatants were collected, and TNF-α levels were measured by sandwich enzyme-linked immunosorbent assay (ELISA). Data are representative of three independent experiments performed in triplicates and are expressed as mean ± standard error of the mean (SEM) (*p < 0.01). **(b)** NBD peptide blocks TNF-α-induced IL-6 production of RA synovial biopsies *ex vivo*. Synovial biopsies were cultured with TNF-α (10 ng/ml) in the presence or absence of NBD peptides (100 μM). After 7 days, supernatants were collected and IL-6 levels were measured by sandwich ELISA and corrected for weight of the biopsy. Data are representative of three independent experiments performed in triplicates and are expressed as mean ± SEM (*p < 0.01).

To gain more knowledge on the therapeutic potential of the NBD peptide in humans, we extended the experiments to another pivotal cell type in the pathogenesis of RA, the macrophage. We found that NBD treatment of human macrophages results in significantly reduced IL-1-β-induced TNF-α production compared with MUT-treated macrophages (*p *< 0.01) (Figure 5a). Finally, we conducted true translational research in which we evaluated the effects of our highly specific IKK-β inhibitor on human synovial tissue. Therefore, we collected synovial biopsies from patients with RA by arthroscopy and cultured the biopsies in the presence or absence of the NBD peptide, followed by TNF-α stimulation. TNF-α was chosen for stimulation of whole-tissue synovial biopsies because this cytokine has been demonstrated to be pivotal in the pathogenesis of RA (reviewed in [30]). Supernatants were collected, and IL-6 production was measured by ELISA. NBD treatment resulted in a significant reduction of TNF-α-induced IL-6 production compared with MUT treatment or TNF-α stimulation alone (2.99 ± 0.01 versus 5.18 ± 0.61 or 6.40 ± 0.22; *p *< 0.01) (Figure 5b). In line with previous observations [17,18], no effect of the NBD peptides on basal IL-6 production was observed (data not shown), because the NBD peptide selectively blocks the induction of NF-κB activity in response to pro-inflammatory stimuli without affecting basal NF-κB activity [20]. In conclusion, these experiments demonstrate the effectiveness of the NBD peptide in human cells.

## Discussion

In the present study, we show for the first time that i.a. administration of the highly specific IKK-β inhibitor NBD peptide significantly reduces arthritis activity and bone destruction *in vivo*. These results indicate that IKK-β-targeted NF-κB inhibition using selective pharmacological inhibitors is beneficial in the local treatment of established arthritis. Of note, only two i.a. injections with the NBD peptide resulted in sustained reduction of the severity of arthritis in a therapeutic setting. Consistent with these observations, synovial inflammation was decreased as demonstrated by a decline in synovial cellularity and reduced levels of the pro-inflammatory cytokines TNF-α and IL-1-β. Importantly, i.a. NBD treatment concomitantly resulted in reduced bone destruction, in agreement with the effects shown after systemic treatment in murine collagen-induced arthritis [18].

The biological effects of local NBD treatment are also consistent with those observed using a gene therapy approach to target IKK-β locally in the joint [16]. Selective pharmacological NF-κB inhibitors may reach the clinic faster because of possible safety and dose regulation issues that accompany gene therapy. However, some of these issues might be resolved by using vectors optimized for i.a. use (for example rAAV5 [31] and disease-inducible promotors or other regulatable gene expression systems [32]).

In RA, FLS and macrophages play important roles in the perpetuation of synovial inflammation [5,6]. Our results indicate that the NBD peptide may have great potential in humans as well, because this NF-κB inhibitor efficiently blocked IL-1-β-induced IκBα phosphorylation and IL-6 production in RA FLS, as well as TNF-α production by human macrophages. One of the important advantages of the NBD peptide, compared with other IKK inhibitors, is that basal NF-κB activity remains unaffected while NF-κB activation in response to pro-inflammatory stimuli is effectively blocked [20]. Therefore, the beneficial role of NF-κB in normal cellular functions is preserved, resulting in less toxicity. Consequently, the effects of the NBD peptide on pro-inflammatory cytokine production *in vitro *were not due to increased apoptosis or necrosis (data not shown). In addition, TNF-α-induced pro-inflammatory cytokine production in cultured synovial biopsies from patients with RA was also significantly reduced. In these synovial biopsies, the micro-architecture of the synovium is preserved, allowing investigators to study the effects of the NBD peptide on synovial inflammation in the complex, biologically relevant network of cells that contribute to the inflammatory process rather than in individually cultured cell types. Thus, this may serve as a model to predict a possible therapeutic effect in human disease [33].

Many anti-inflammatory drugs used in the treatment of arthritis target, at least in part, NF-κB. Of these drugs, glucocorticoids like dexamethasone and prednisolone (although non-specific) are considered the most powerful NF-κB inhibitors [34,35]. Intra-articular steroid injections are widely used to control local inflammation. In addition to showing local side effects such as reduced bone formation [36], recent work has shown unwanted systemic effects due to absorption of steroids from the i.a. space [37,38]. The most common side effect caused by systemic absorption of i.a. steroids is suppression of pituitary-adrenal axis function [38]. This suppression may last from up to 2 weeks to even 6 months after i.a. injection and may ultimately lead to adrenal failure [39]. Therefore, there is a clear need for potent anti-inflammatory drugs for i.a. administration without steroid action to prevent these unwanted side effects. Pharmacological NF-κB inhibitors like the NBD peptide may fulfill this need; because they are mainly peptide-based and not steroid-based, they selectively block NF-κB activity in the joint without causing these side effects. In addition, the NBD peptide inhibits only pro-inflammatory IKK activity [20] and may therefore be safer than other IKK inhibitors if absorbed from the i.a. space and released systemically. However, extensive pharmacological evaluation of this approach is required to carefully monitor pharmacokinetics and pharmacodynamics, as well as potential toxicity, of new pharmacological NF-κB inhibitors like the NBD peptide before clinical trials with these compounds may be initiated.

## Conclusion

We have demonstrated that local small peptide-mediated NF-κB inhibition not only ameliorated established arthritis and reduced bone destruction in an animal model of RA, but also prevented pro-inflammatory cytokine production by human RA synovial biopsies. Our results suggest that i.a. treatment with the NBD peptide may represent a novel therapeutic approach in RA.

## Abbreviations

AA = adjuvant arthritis; AUC = area under the curve; BSA = bovine serum albumin; DMEM = Dulbecco's modified Eagle's medium; ELISA = enzyme-linked immunosorbent assay; FCS = fetal calf serum; FLS = fibroblast-like synoviocytes; HRP = horseradish peroxidase; i.a. = intra-articular; IκB = inhibitor of κB; IKK = IκB kinase; mAb = monoclonal antibody; MOD = mean optical density; MUT = mutant nuclear factor-κB essential modulator binding domain peptide; NBD = nuclear factor-κB essential modulator binding domain peptide; NEMO = nuclear factor-κB essential modulator; NF = nuclear factor; PBS = phosphate-buffered saline; ph = phosphorylated; RA = rheumatoid arthritis; TBS = Tris-buffered saline; TNF = tumor necrosis factor.

## Competing interests

The authors declare that they have no competing interests.

## Authors' contributions

SWT carried out Western blots, ELISAs, and animal studies, evaluated radiological scores and immunohistochemical stainings, and drafted the manuscript. MJV participated in the design of the study, evaluated radiological scores, and helped to draft the manuscript. NH assisted in the animal studies and cell culture and performed immunohistochemical stainings and digital image analysis. MJM and SG participated in the design of the study and helped to draft the manuscript. PPT conceived of the study, participated in its design and coordination, and helped to draft the manuscript. All authors read and approved the final manuscript.
